# Is Endoscopic Approach Alone Adequate for the Management of Ureterovaginal Fistulas?

**DOI:** 10.7759/cureus.85710

**Published:** 2025-06-10

**Authors:** Saurabh Kumar, Amit Jain, Sameer Vyas, Narendra Kurmi, Saurabh Jain, Ajaybhai Jani, Skandh Bhatia

**Affiliations:** 1 Department of Urology, Gandhi Medical College, Bhopal, IND

**Keywords:** dj stent, endoscopic management, minimally invasive, ureteric stent, ureterovaginal fistula

## Abstract

Background: Most ureterovaginal fistulas (UVFs) are caused by gynecologic, urologic, or colorectal surgeries. Urine leaks, renal failure, and infections lower patients' quality of life. Minimally invasive endoscopic double-J (DJ) stenting has become popular. There is insufficient research on the effects of DJ stenting on fistula size, diagnostic timeliness, and patient comorbidities.

Objective and methods: This study examines the efficacy of endoscopic DJ stent implantation in treating UVFs and addresses aspects such as fistula size, diagnosis timing, and comorbidities. This is a five-year retrospective study (2019 to 2024) conducted in Bhopal, India, comprising 31 patients with UVF who received endoscopic DJ stenting as the main treatment. Analyses included patient demographics, clinical presentation, fistula features, treatment outcomes, and complications. Statistical analysis includes chi-square tests for categorical variables and logistic regression for risk factor assessment, with a p-value < 0.05 considered significant.

Results: DJ stenting showed a success rate of 77.4% (24/31 cases), with higher rates for early diagnosis (<4 weeks) and small fistula size (<5 mm) (p=0.038 and 0.032, respectively). Late diagnosis (>4 weeks), large fistula size (>5 mm), diabetes, and elevated creatinine (>1.2 mg/dL) were independent predictors of treatment failure in multivariate analysis. Minor issues included dysuria (16.1%, n=5) and hematuria (9.7%, n=3). One patient (3.2%) needed surgery due to a forgotten DJ stent.

Conclusion: If the UVF is minor and detected early, endoscopic DJ stenting can work. Renal failure, diabetes, larger fistulas, and delayed diagnosis reduce treatment success. Early prognostic identification and patient selection are crucial to maximize results and minimize surgery.

## Introduction

Ureterovaginal fistula (UVF) is an abnormal communication between the ureter and vagina, leading to continuous vaginal urine leakage [1]. It commonly occurs as a complication of gynecologic, urologic, or colorectal surgeries, with total abdominal hysterectomy (TAH) being the most frequent cause [2]. Other causes include pelvic radiation therapy, cesarean sections, radical hysterectomy, and vascular procedures, often resulting from inadvertent ureteral injury during surgery [3]. Such injuries, including thermal damage, transection, or ligation, lead to significant morbidity, affecting a patient’s quality of life due to persistent urinary incontinence, recurrent infections, and progressive renal dysfunction [4].

While open surgical repair (such as ureteroneocystostomy, ureteroureterostomy, psoas hitch, and Boari flap) is considered the gold standard for managing UVF, these procedures are invasive, require prolonged hospitalization, and carry risks of complications such as ureteral strictures and infections; also, they are not suitable for further endoscopic procedures in the future [5]. Minimally invasive alternatives, particularly endoscopic double-J (DJ) stenting, have been proposed as a primary management strategy [6]. The stent functions by reducing the pressure gradient across the ureteral defect, promoting fistula closure while ensuring effective urinary drainage [7]. However, there is limited consensus on the effectiveness of DJ stenting as a primary treatment, particularly regarding its success rates concerning fistula size and the timing of diagnosis [8]. Early intervention and smaller fistulas increase success rates, while stenting alone may not be enough for bigger or chronic fistulas [9]. This knowledge gap requires further research into DJ stenting for UVF success factors.

This study conducted in Bhopal, India, evaluates endoscopic DJ stent implantation as a primary UVF treatment and investigates its success factors. We studied fistula size and diagnosis timing on treatment outcomes. This study analyzed real-world clinical data to determine whether endoscopic care can reduce the need for more invasive surgery. We expect our findings will fill the study gap and improve UVF treatment techniques.

## Materials and methods

Study design and setting

This five-year retrospective study included medical records of patients with UVF from 2019 to 2024. The study investigated endoscopic double-J stenting for UVF treatment. Pre-data collection consent was received from the Institutional Ethics Committee, Gandhi Medical College (Bhopal, MP, IND), and de-identification of medical records protected patient confidentiality and human subjects. Statistical analysis includes chi-square tests for categorical variables and logistic regression for risk factor assessment, with a p-value < 0.05 considered significant.

Patient selection criteria

**Figure 1:**
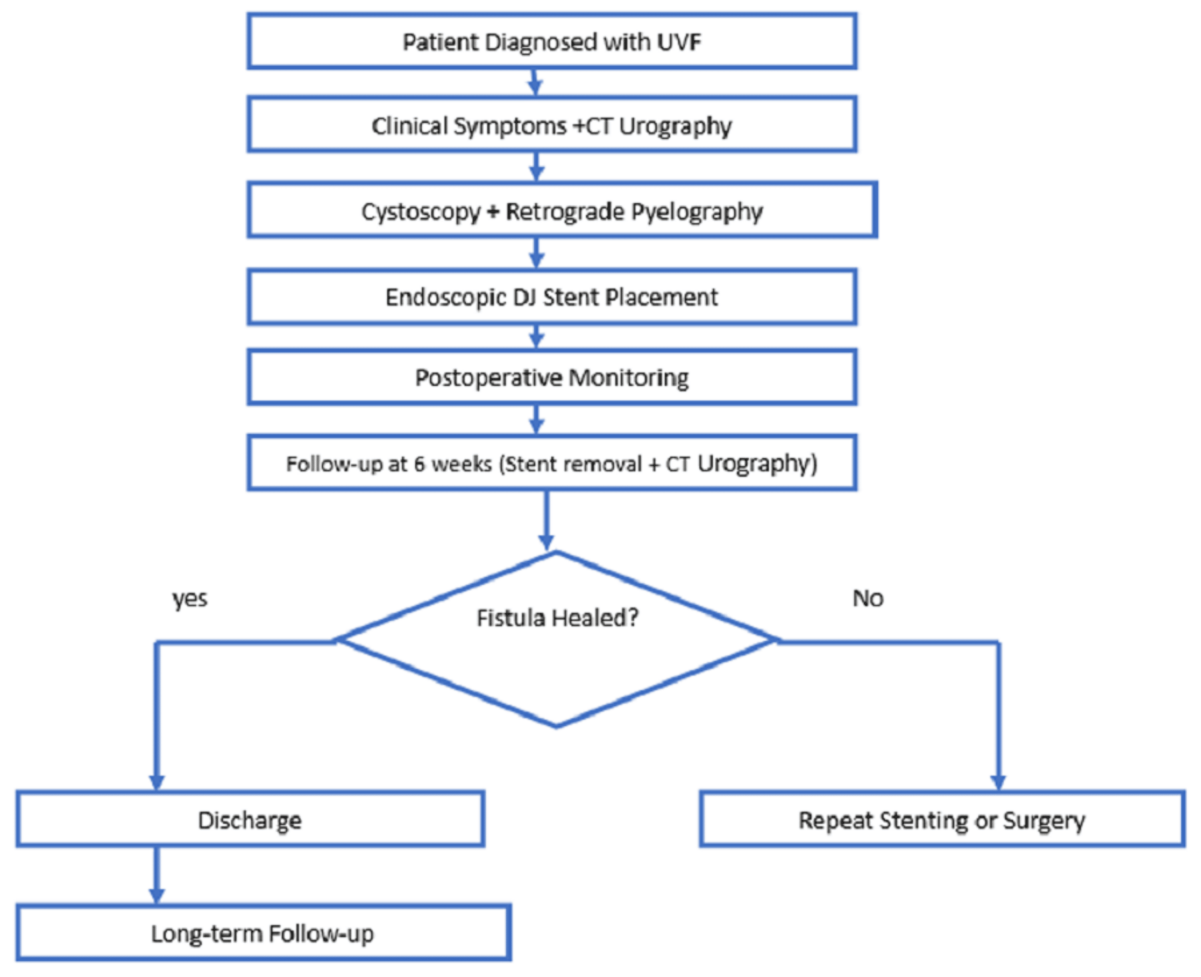
Diagnostic evaluation, treatment protocol and follow up algorithm of the study

This study included 31 radiologically and endoscopically verified UVF patients. Patients with UVF were diagnosed through clinical symptoms and radiological confirmation (CT urography and retrograde pyelography). Patients who underwent endoscopic DJ stent implantation for primary management and patients without past UVF procedures before DJ stent implantation were included in the study. However, patients requiring immediate open surgical repair due to extensive ureteral injury, patients with complex urinary tract abnormalities necessitating reconstructive surgery, and patients with severe ureteral strictures preventing successful endoscopic stent placement were excluded. The diagnostic evaluation, treatment protocol, and follow-up algorithm of the study are depicted in Figure 1.

Diagnostic evaluation

**Figure 2:**
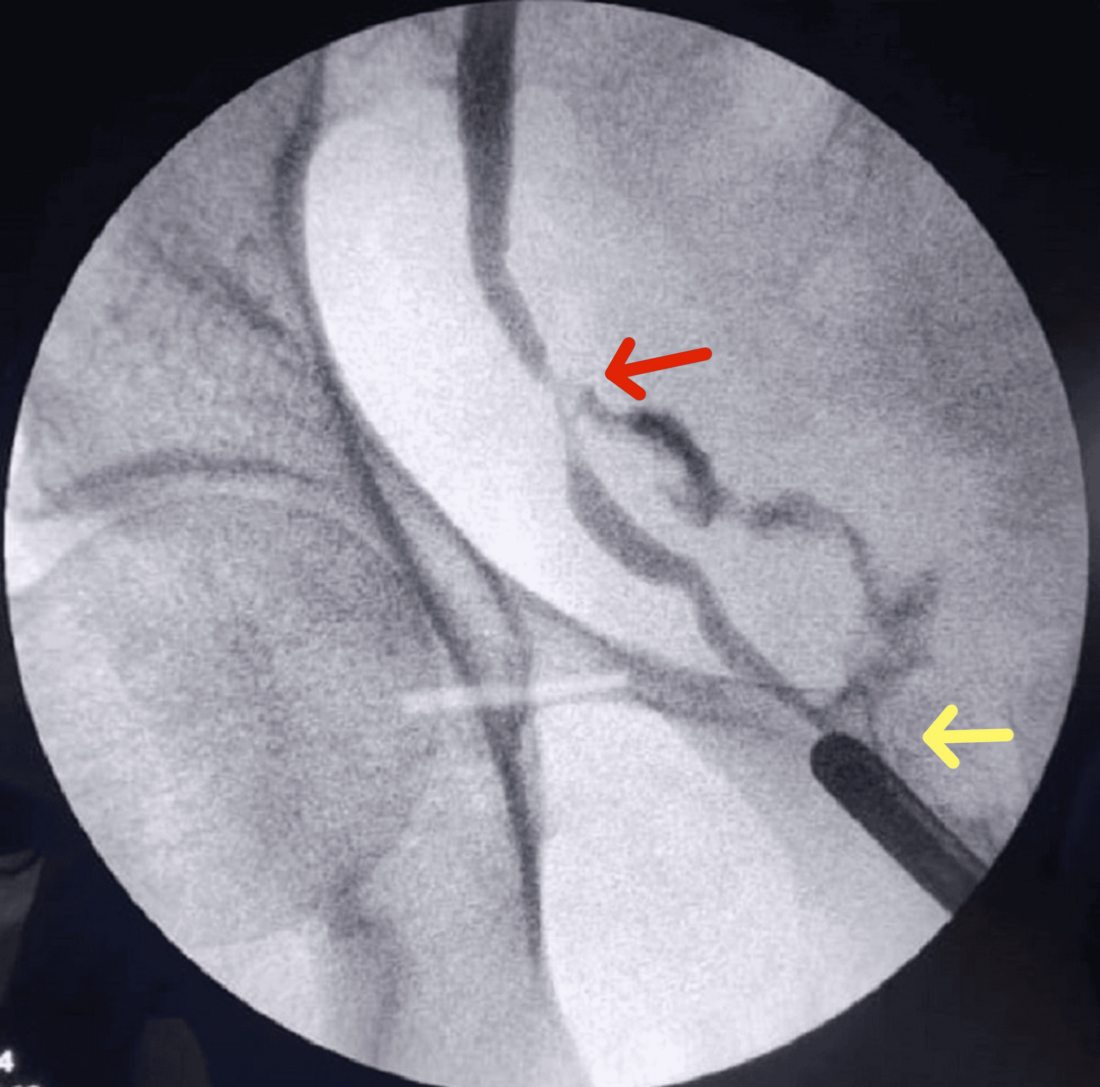
Retrograde pyelography shows contrast extravasation (red arrow) and UVF (yellow arrow) UVF: Ureterovaginal fistula

**Figure 3:**
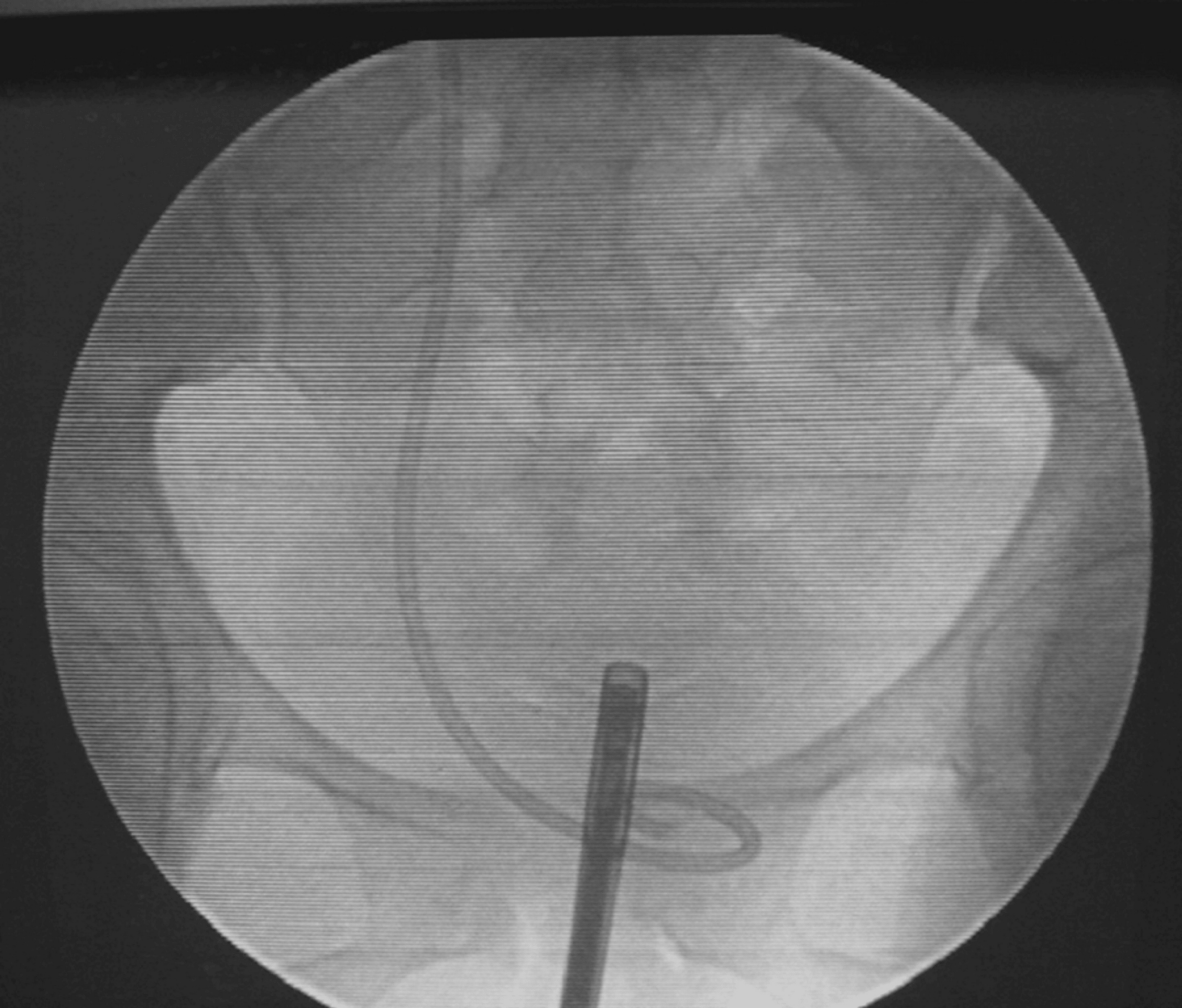
Placement of DJ stent

The CT urography was performed in all patients to confirm the presence of UVF, its location, and any hydronephrosis or ureteral obstruction. A cystoscopy check was done for bladder fistulas. Intraoperative retrograde pyelography pinpointed the fistula and guided the DJ stent placement (Figures 2-3), ensuring precise treatment planning.

Treatment protocol

The affected ureter was retrogradely implanted with a 5 Fr DJ stent under fluoroscopic guidance. After six weeks, the stent closed the fistula by reducing pressure and maintaining urine drainage. Patients were monitored for pain, hematuria, and infection after surgery. Antibiotics and hydration were given during recovery to prevent stent encrustation and infections.

Follow-up schedule

After six weeks, the DJ stent was removed by cystoscopy, and a CT urography was done to check for fistula closure. Successful closure was indicated by no urine leakage and no residual fistula on imaging. Patients with unclosed fistulas were re-evaluated for DJ stenting or surgery. Patients were evaluated for ureteral strictures and recurrent fistulas during long-term follow-up to ensure full assessment and care. 

## Results

Patient demographics and clinical characteristics

The average age of the 31 patients in the study was 46.2 ± 8.4 years, based on demographic and clinical data (Table 1). Around 77.4% (n=24)of women had UVFs after hysterectomy. In addition, 9.7% (n=3) suffered pelvic cancer fistulas and 12.9% (n=4) had radiation complications. The metabolic profile was stable, with mean hemoglobin and albumin values of 11.8 ± 2.1 g/dL and 3.9 ± 0.6 g/dL, respectively. With a mean BMI of 25.3 ± 3.2 kg/m², most patients were classed as overweight. Diabetes mellitus was present in 29.0% (n=9) and previous stone surgery was seen in 22.6% (n=7) patients.

**Table 1:** Patient demographics and clinical characteristics UVF: Ureterovaginal fistula

Parameter	Number (total n=31)
Age (Mean ± SD)	46.2 ± 8.4
Hemoglobin (g/dL, Mean ± SD)	11.8 ± 2.1
Albumin (g/dL, Mean ± SD)	3.9 ± 0.6
BMI (kg/m², Mean ± SD)	25.3 ± 3.2
Diabetes mellitus (yes)	9 (29.0%)
Previous stone surgery (yes)	7 (22.6%)
Creatinine (mg/dL, mean ± SD)	1.2 ± 0.3
Post-hysterectomy UVF	24 (77.4%)
Pelvic malignancy	3 (9.7%)
Radiation therapy	4 (12.9%)

Symptoms and clinical presentation 

The predominant symptom was continuous urinary leakage (90.3%, n=28), followed by flank pain (35.5%, n=11) and recurrent urinary tract infection (UTI) (19.4%, n=6). The most common symptom described by patients who underwent a hysterectomy (n=24) and those who did not (n=7) was continuous urine leakage, which was present in 91.7% (n=22) of post-hysterectomy cases and 85.7% (n=6) of non-hysterectomy cases. These findings show that UVF is mainly characterized by chronic urine leakage (Table 2).

**Table 2:** Symptoms and clinical presentation UTI: Urinary tract infection

Symptom	Post-hysterectomy (n=24)	Non-hysterectomy (n=7)
Continuous urinary leakage	22 (91.7%)	6 (85.7%)
Flank pain	9 (37.5%)	2 (28.6%)
Recurrent UTI	5 (20.8%)	1 (14.3%)

Factors influencing treatment success 

The examination of these aspects revealed many key elements that affect the effectiveness of DJ stenting in treating UVFs (Table 3). Early detection (<4 weeks) was related to a higher success rate (92.9%, n=13/14), while patients treated after four weeks had a lower success rate (64.7%, n=11/17; p=0.038). Endoscopic therapy had a success rate of 89.5% (n=17/19) for minor fistulas (<5 mm) and 58.3% (n=7/12) for larger ones (>5 mm) (p=0.032). Five of the nine diabetic patients (55.6%) needed extra intervention due to persistent fistula, and diabetes mellitus was significantly associated with treatment failure (p=0.043). The minor but significant link between past stone surgery and failure (p=0.051) suggests that scarring or structural defects from previous ureteral surgeries may affect healing. Failure rates were significantly correlated with creatinine levels (>1.2 mg/dL; p = 0.047), suggesting renal dysfunction may harm fistula closure and recovery. Early detection, smaller fistulas, and no severe comorbidities like diabetes or renal insufficiency make DJ stenting in UVF therapy effective. Patients with larger fistulas, late diagnoses, and renal impairment may need additional treatments.

**Table 3:** Factors influencing treatment success *p<0.05 considered significant; NA: Not available

Factors	Success rate (n=24)	Failure rate (n=7)	p-value
Early diagnosis (<4 weeks)	13	1	0.038*
Late diagnosis (>4 weeks)	11	6	NA
Small fistula (<5mm)	17	2	0.032*
Large fistula (>5mm)	7	5	NA
Diabetes present	4	5	0.043*
Previous stone surgery	2	5	0.051
Creatinine (>1.2 mg/dL)	3	5	0.047*

Univariate and multivariate analysis

Univariate logistic regression research identified several characteristics that could predict treatment failure for DJ-stented UVFs (Table 4). Delays in diagnosis decreased the likelihood of spontaneous fistula closure: >4 weeks (OR = 3.2, 95% CI: 1.1-7.6, p = 0.028). The likelihood of failure was 4.1 times greater for fistulas larger than 5 mm (95% CI: 1.5-8.3, p = 0.015). Diabetics had a higher rate of treatment failure (95% CI: 1.2-9.1, p = 0.031). Prior stone surgery was associated with failure (OR = 3.5, 95% CI: 0.98-7.8, p = 0.058), but statistical significance was not shown. Results suggested that renal dysfunction hinders fistula resolution. Failure is strongly predicted with renal impairment (creatinine levels >1.2 mg/dL, OR = 4.0, 95% CI: 1.3-8.9, p = 0.021). Our results emphasize the importance of early identification, small fistula size, and stable renal function for optimal treatment. Patients with delayed diagnosis, larger fistulas, diabetes, or renal impairment may need additional or complementary surgeries to improve results.

**Table 4:** Univariate analysis (risk factors for treatment failure) *p<0.05 considered significant

Variable	Odds ratio (95% CI)	p-value
Late diagnosis (>4 weeks)	3.2 (1.1–7.6)	0.028*
Large fistula (>5mm)	4.1 (1.5–8.3)	0.015*
Diabetes mellitus	3.8 (1.2–9.1)	0.031*
Previous stone surgery	3.5 (0.98–7.8)	0.058*
Creatinine (>1.2 mg/dL)	4.0 (1.3–8.9)	0.021*

Multivariate analysis (stepwise regression for independent risk factors)

Multivariate logistic regression research identified four characteristics linked with treatment failure for DJ-stented UVF (Table 5). Delays in diagnosis (>4 weeks) were revealed to be a significant risk factor (adjusted OR=2.9, 95% CI: 1.1-6.5, p=0.033), showing that delayed intervention considerably reduces the likelihood of fistula closure. The chance of failure was 3.7 times higher for fistulas larger than 5 mm (95% CI: 1.4-7.9, p=0.019), indicating that larger defects might require multiple surgeries. Diabetes and other comorbidities still predicted failure (adjusted OR=2.6, 95% CI: 1.1-6.3, p=0.047). Treatment failure was also associated with creatinine levels >1.2 mg/dL (adjusted OR=3.4, 95% CI: 1.3-7.8, p=0.026), highlighting the importance of renal dysfunction on fistula healing. As indicated in these data, DJ stenting success depends on early identification, smaller fistula size, and sustained renal function. Patients with large fistulas, diabetes, renal impairment, or delayed diagnosis may need closer monitoring, lengthier stenting, or surgery to improve treatment outcomes.

**Table 5:** Multivariate analysis (independent risk factors for failure) *p<0.05 considered significant

Independent predictor	Adjusted odds ratio (95% CI)	p-value
Late diagnosis (>4 weeks)	2.9 (1.1–6.5)	0.033*
Large fistula (>5mm)	3.7 (1.4–7.9)	0.019*
Diabetes mellitus	2.6 (1.1–6.3)	0.047*
Creatinine (>1.2 mg/dL)	3.4 (1.3–7.8)	0.026*

Complications and follow-up

There were no major intraoperative complications. Complications and follow-up outcomes in 31 DJ stenting patients for UVF patients are shown in Table 6. Minor complications occurred in 16.1% of individuals (n=5), with dysuria being the most common and hematuria in 9.7% (n=3). These symptoms resolved themselves after a week or two; thus, no therapy was needed. After two years, one patient (3.2%) developed recurrence due to a forgotten DJ stent that formed a chronic fistula and required ureteric reimplantation. The DJ stenting manages UVF less invasively and has a low recurrence rate under ideal settings. These findings underscore the importance of regular follow-up to prevent stent retention and recurrence.

**Table 6:** Complications and follow-up outcomes

Complication	Number (total n=31)	Percentage
Dysuria	5	16.1%
Hematuria	3	9.7%
Major complications	0	0%
Recurrence (follow-up)	1	3.2%

## Discussion

Interpretation of key findings

This study highlights the efficacy of endoscopic DJ stenting as a minimally invasive treatment for UVFs, with a success rate of 77.4% (n=24). The key predictors of successful treatment included early diagnosis (<4 weeks) and small fistula size (<5 mm). Patients diagnosed within four weeks had a significantly higher success rate (92.9%, n=13/14)) compared to those diagnosed later (64.7%, n=11/17, p = 0.038). Similarly, patients with smaller fistulas had a higher success rate (89.5%, n=17/19) than those with larger defects (58.3%, n=7/12, p=0.032). Conversely, diabetes mellitus, prior stone surgery, and renal dysfunction (creatinine >1.2 mg/dL) were associated with higher failure rates, emphasizing the importance of patient selection. Minor complications such as dysuria (16.1%, n=5) and hematuria (9.7%, n=3) were observed but resolved without intervention. One patient (3.2%) developed a chronic fistula due to a retained stent, necessitating surgical repair. These findings suggest that endoscopic DJ stenting is a viable first-line treatment for UVF in select patients, particularly when early intervention is possible.

Comparison with existing literature

The results of this study align with prior research that supports endoscopic management as a viable alternative to open surgery in specific cases. A study by Lo et al. [10] demonstrated that early detection and smaller fistulas significantly improve treatment success rates. Similarly, Kajabwangu et al. [11] reported that delayed diagnosis reduces the effectiveness of endoscopic management, leading to higher rates of surgical intervention. However, other studies emphasize the limitations of DJ stenting, particularly for larger and chronic fistulas. Sharma and Chaudhary [12] reported that failure rates increase in cases where the fistula exceeds 5 mm or persists beyond four weeks. This study’s findings reinforce that while endoscopic DJ stenting is effective, it is not a universal solution, particularly in cases complicated by diabetes or renal impairment. The results also confirm previous reports that higher creatinine levels negatively impact fistula closure, as demonstrated in the study by Deng et al. [13]. In our study, the success rate of DJ stenting for UVF is 77.4% (n=24/31), while the study by Al Otaibi [4] reported a success rate of 64% (n=7/11), which might be due to the small sample size. So, studies with a larger sample size will be needed to conclude the outcome of DJ stenting in UVF patients. Chen et al. [3] concluded that DJ stenting is associated with minimum morbidity and delayed treatment is a risk factor for stent failure, but not an absolute contraindication, which aligns with our study. Chen et al. [3] also recommended an attempt at DJ stenting in all UVFs, despite the timing of diagnosis. Studies with long-term follow-up will be needed to conclude the long-term complications associated with DJ stenting in patients with UVF.

Limitations

Despite the valuable insights gained, this study has several limitations. First, it is a retrospective analysis, which may introduce selection bias. The sample size is relatively small (n=31), limiting the generalizability of the findings. Additionally, the follow-up period was not standardized for all patients, making it difficult to assess long-term recurrence rates comprehensively. Another limitation is the lack of direct comparison with other treatment modalities, such as open surgical repair or robotic-assisted ureteral reimplantation. While DJ stenting was effective in most cases, a comparative study would provide a more definitive assessment of its relative advantages and limitations. Finally, patient comorbidities such as diabetes and renal dysfunction were identified as risk factors, but additional studies with larger sample sizes are needed to quantify their exact impact.

Future recommendations

To further refine the management of UVFs, future research should focus on prospective studies comparing endoscopic DJ stenting with other treatment modalities. Randomized controlled trials assessing different fistula closure techniques would provide stronger evidence regarding the optimal management approach. Additionally, advancements in stent technology could improve outcomes. Drug-eluting stents or bioresorbable stents may enhance fistula healing and reduce recurrence rates. Investigating alternative conservative therapies, such as fibrin glue or tissue engineering techniques, could also be beneficial. Finally, long-term follow-up studies are needed to evaluate recurrence rates, late complications, and overall patient satisfaction. Establishing standardized protocols for patient selection, follow-up intervals, and secondary intervention criteria will further optimize treatment success.

## Conclusions

This study demonstrates that endoscopic DJ stenting is an effective, minimally invasive approach for the management of UVFs, particularly in cases diagnosed early (<4 weeks) and with smaller fistula sizes (<5 mm). The findings highlight that delayed diagnosis, larger fistula size, diabetes mellitus, and elevated creatinine levels (>1.2 mg/dL) significantly reduce the likelihood of successful fistula closure. Given the 77.4% overall success rate, early intervention and careful patient selection based on prognostic factors can optimize outcomes and reduce the need for surgical repair. Additionally, long-term follow-up is essential to monitor for recurrence and complications such as ureteral strictures, ensuring sustained treatment success. Future research should focus on alternative conservative therapies and improved stenting techniques to enhance success rates in high-risk patients.
